# 

**Published:** 1912-11-15

**Authors:** 


### ANNOUNCEMENTS

Officers Arizona Dental Society. President, J. A. Messinger; Vice-President, L. W. Downs; Secretary-Treasurer, H. H. Wilson. The annual meeting was held in Phoenix October 29th to 31st 1912.

Correction.—In our October issue we reprinted an article by Dr. J. O. Eppright, on the technique of making a porcelain jacket crown, which should have been credited to the *Dental Review,* and not to the *Dental Record* as the printer made it appear, in spite of the fact that we clearly gave the proper credit in our copy. Our apologies are made to the Review.

Minneapolis Dental Society—The January meeting of this Society will be held January 17th and 18th, 1913, as the date announced was found to conflict another society. Please make a note of the change.

O. D. Davis, Sec'y.

Indiana State Dental Association. The fifty-fifth annual session of the Indiana State Dental Association will be held at the Claypool Hotel, Indianapolis, May 20, 21, 22, 1913.

The officers of the association recently met at Indianapolis and perfected plans for a three-days "Post Graduate Course." The very best instructors and specialists are being secured for each day.

The course will be as follows: Tuesday, "Humanitarian

Dentistry," Wednesday, "Preventive Dentistry," Thursday, A. M. "Prosthodontia," Thursday P. M., a great table clinic; the clinic will also be held in the hotel.

No tuition fee for the members of the Association, or visitors from outside the State who are in good standing in their State Association, but all others desiring to take this course must arrange their tuition fees with the secretary.

OTTO U. KING, *Sec'y.*

MINNEAPOLIS DENTAL SOCIETY. The annual mid-winter meeting of the Minneapolis Dental Society will be held in the Masonic Temple, Minneapolis, Minn., on Friday and Saturday January 24 and 25, 1913.

Space has already been reserved for even a larger Manufacturers' exhibit than was given last year.

Clinics will be given by some of the best men in the profession who will demonstrate all the newest and most useful methods.

The entire meeting is to be conducted along unique and original lines,—a new method of arranging the exhibits; also many other new things which will add to the pleasure and profit of each visitor.

For information address:
O. DEFOREST DAVIS, *Sec'y.*
404 Donaldson Bldg.,
Minneapolis, Minn.

OHIO STATE DENTAL MEETING. The Committees appointed to prepare for the holding of the 47th Annual Meeting of the Ohio State Dental Society, at Cincinnati, December 3, 4, 5, 1912, have been and are diligently carrying on their work.

The program as it stands at present is both unique and interesting.—

| | | |
|---|---|---|
| President's Address, | Dr. C. R. Converse, | Springfield. |
| Professional Ethics, | H. C. Brown, | Columbus. |
| Oral Surgery, | J. V. I. Brown, | Milwaukee, Wis. |
| Removable Bridge Work, | Chas. F. Ash, | New York City. |
| Instrumentation in Pyorrhea Treatment. | A. F. James, | Chicago, Ills. |
| The Diagnosis of Diseases of the Pulp, | Herman Prinz, | St. Louis, Mo. |

Lecture, Venereal Diseases, Illustrated by stereopticon and moving pictures from actual subjects, by A. E. Deeds, Dayton, Ohio.

This lecture has been prepared by the National Cash Register Co., at a cost of about $15,000. The Medical Societies before which it has been presented pronounce it a wonderful exhibit. It is needless to call attention to the growing necessity for a thorough understanding of these diseases by the Dental Profession.

The local Oral Hygiene committee will give a practical exhibition of their school work, by having a whole school at work at their own school building, under the direction of The Oral Hygiene Committee of the Cincinnati Society, giving every detail of the work, from primary examinations up to operative work.

The practical working of all the latest phases of Dental Radiography will be demonstrated by Dr. Sidney Lange of Cincinnati. One of the most complete outfits will be set up and radiogrames taken and developed and finished and delivered on the spot.

The Clinics will be very attractive.

The Hotel Sinton will be the Headquarters.

F. R. Chapman, *Sec'y*,
Columbus, O.

